# Antiepileptic Strategies for Patients with Primary and Metastatic Brain Tumors

**DOI:** 10.1007/s11864-024-01182-8

**Published:** 2024-02-14

**Authors:** Herbert B. Newton, Jenna Wojkowski

**Affiliations:** 1 Neuro-Oncology Center and Brain Tumor Institute, University Hospitals of Cleveland Medical Center, Seidman Cancer Center, Hanna Hall 5th Floor, 11100 Euclid Avenue, Cleveland, OH 44106 USA; 2 Department of Pharmacy, University Hospitals of Cleveland Medical Center, Seidman Cancer Center, Cleveland, OH USA; 3 Present Address: Department of Pharmacy, Duke University Medical Center, Durham, NC USA

**Keywords:** Brain tumors, Gliomas, Seizures, Brain tumor–related epilepsy, Glioblastoma, IDH mutation, Epileptogenesis, Levetiracetam, Lacosamide, Valproic acid

## Abstract

Seizure activity is common in patients with primary and metastatic brain tumors, affecting more than 50% of cases over the course of their disease. Several mechanisms contribute to brain tumor–related epilepsy (BTRE), including a pro-inflammatory environment, excessive secretion of glutamate and an increase in neuronal excitatory tone, reduction of GABAergic inhibitory activity, and an increase in 2-hydroxygluturate production in isocitrate dehydrogenase mutant tumors. After a verified seizure in a brain tumor patient, the consensus is that BTRE has developed, and it is necessary to initiate an antiepileptic drug (AED). It is not recommended to initiate AED prophylaxis. Second- and third-generation AEDs are the preferred options for initiation, due to a lack of hepatic enzyme induction and reduced likelihood for drug-drug interactions, especially in regard to neoplastic treatment. The efficacy of appropriate AEDs for patients with BTRE is fairly equivalent, although some data suggests that levetiracetam may be slightly more active in suppressing seizures than other AEDs. The consensus among most Neuro-Oncology providers is to initiate levetiracetam monotherapy after a first seizure in a brain tumor patient, as long as the patient does not have any psychiatric co-morbidities. If levetiracetam is not tolerated well or is ineffective, other appropriate initial AED options for monotherapy or as an add-on anticonvulsant include lacosamide, valproic acid, briviracetam, lamotrigine, and perampanel.

## Introduction

Seizure activity is a common occurrence for patients with a primary or metastatic brain tumor. In this setting, the presence of persistent seizure activity is termed Brain Tumor-Related Epilepsy (BTRE) and is variable depending on specific aspects of the tumor, including histology, grade, and molecular phenotype [1–5, 6•]. Epilepsy from a brain tumor constitutes 6–10% of all cases of epilepsy as a whole, and 12% of acquired epilepsy. In general, the incidence of BTRE is higher in low-grade tumors in comparison to high-grade tumors. For example, patients with dysembryoplastic neuroepithelial tumors (DNETs; grade 1) experience a very high seizure incidence at presentation, roughly 100%, while those with other low-grade glioneuronal tumors have an incidence in the 70–80% range. In patients with isocitrate dehydrogenase (IDH) mutant grade 2 diffuse gliomas (including astrocytomas and oligodendrogliomas), the incidence of BTRE is roughly 65–75%. In contrast, the incidence of BTRE in patients with high-grade gliomas ranges from 25 to 60%, including those with IDH wildtype grade 4 glioblastoma (GBM), where the incidence is 25–30%. For patients with meningiomas, the incidence of BTRE is 30–50%, while for those with brain metastases, it is in the 20–35% range.

The seizures in BTRE are usually focal motor events affecting the contralateral face, arm, hand, or leg; or a combination of these locations [1–5, 6•]. Focal seizure activity can also affect speech (e.g., speech arrest). Partial complex-type seizures can also occur if the tumor is located in the anterior temporal lobe. Status epilepticus and non-convulsive status epilepticus can potentially arise in the setting of BTRE, but are not very common. If seizure activity is suspected in a brain tumor patient, but is not totally clear based on the history, then a workup will be necessary [7]. The differential diagnosis for a seizure event is very broad and includes syncope of cardiac origin, syncope of non-cardiac origin, toxic disturbances, and metabolic disturbances. A cardiac workup is often necessary in this situation, along with new imaging studies (MRI, MRS), toxic and metabolic lab studies, baseline EEG, and possibly long-term monitoring in an epilepsy unit.

## Update on epileptogenesis of BTRE

In recent years, it has been clarified that brain tumor–related seizure events do not arise from within the bulk of the tumor tissue, but instead in the peritumoral regions around the tumor, where multiple factors contribute to an ongoing and escalating epileptogenic environment [1, 2, 8, 9, 10•, 11]. The initial alterations to the peritumoral region involve mechanical factors as the tumor enlarges, including compression, regional ischemia, metabolic changes such as acidosis, focal disruption of the blood-brain barrier (BBB) with fluid and protein leakage, hypoxia, glial swelling, and tissue damage—all of which contribute to the early phase of epileptogenesis [8, 9, 10•, 11]. Concomitant with these mechanical alterations are numerous other regional changes related to tumor cells, interactions with neurons, inflammatory processes, molecular factors, receptor activity, and neurotransmitter imbalance.

The peritumoral region is in a pro-inflammatory state, with recruitment of astrocytes, microglial cells, and macrophages, increased concentrations of cytokines including interleukins (IL) IL-1β, IL-6, and IL-8, tumor necrosis factor (TNF)-α, chemokines, and matrix metalloproteinases (MMP) MMP-2 and MMP-9—all of which promote tumor proliferation, invasiveness, and seizure susceptibility [8, 9, 10•, 11]. Within this background, it is also known that glioma cells secrete large amounts of the excitatory neurotransmitter glutamate, which is important in favoring epileptogenesis [6•, 7–9, 10•]. Secretion of glutamate is primarily mediated by the xCT cysteine/glutamate antiporter, which has increased expression on the surface of glioma cells and is an independent biomarker for seizures in these patients [12, 13]. In addition, peritumoral astrocytes demonstrate impaired expression of excitatory amino acid transporter 1 (EAAT1) and EAAT2, which uptake synaptic glutamate, thereby contributing to the accumulation of glutamate in the extracellular space. In parallel with an increased availability of glutamate, there is an augmented expression of ionotropic α–amino-3-hydroxy-5-methyl-4-isoxazolepropionic acid (AMPA) and N-methyl-D-aspartate (NMDA) receptors in glioma cells and peritumoral astrocytes, which contributes to the high glutamatergic tone and excitability in the peritumoral region [12]. Stimulation of AMPA and NMDA receptors by glutamate promotes growth and invasiveness in glioma cells; however, for neurons, it promotes epileptogenesis and excitotoxicity and may lead to neuronal death. In addition, it has been shown that glioma cells can form microtubes—thin tube-like structures with membranes—that can develop functional synapses with nearby neurons (i.e., neurogliomal synapses) that communicate via postsynaptic currents mediated by AMPA-glutamate receptors [8, 9, 10•]. This has led to extensive research into the utility of inhibiting AMPA receptor activity (e.g., perampanel; see below).

Recent research has revealed that the presence of a mutation in IDH can have a direct role in promoting epileptogenesis and is associated with a more aggressive BTRE phenotype in glial tumors, via several different mechanisms [2, 8, 9, 10•, 11, 14, 15]. The IDH mutation results in the conversion of α–ketoglutarate into 2-hydroxyglutarate (2-HG), which accumulates in and is secreted by glioma cells and has structural similarity to glutamate. 2-HG is able to function as a glutamate agonist, further increasing excitatory tone by activating NMDA receptors in surrounding neurons—thereby promoting epileptogenesis. In addition, recent in vitro data from Mortazavi and colleagues [16] suggests that the presence of elevated levels of D-2-HG results in metabolic re-programming of peritumoral neurons, such that they become hyperexcitable—including elevated spiking activity. This alteration of neuronal excitability and metabolic activity is mediated via upregulation of the mTOR signaling pathway.

In parallel with the increase in glutamatergic excitatory activity in the peritumoral region, there is also a reduction of inhibitory γ-aminobutyric acid (GABA) input to neuronal cells, further increasing the potential for epileptogenesis [2, 8, 9, 10•, 11, 12]. There is evidence for altered expression of chloride transporters on the neuronal surface, with an increase in the concentration of Na^+^-K^+^-Cl^−^ cotransporter 1 (NKCC1) and a reduction in the concentration of Cl^−^-K^+^ symporter 5 (KCC2). These alterations result in increased neuronal concentrations of Cl^−^, so that when GABA_A_ receptors are activated there is an efflux of Cl^−^ out of the cell, resulting in a paradoxical depolarization and activation of the neuron [12]. In addition, upregulation of several subunits of the GABA_A_ receptor (α1, α5, β1, β3) on glioma cells may lead to impairment of tonic GABAergic inhibition, and thus increased neuronal excitability.

In addition to the mechanisms reviewed above, other contributors to the process of peritumoral epileptogenesis include reduced expression of glutamine synthetase in regional astrocytes, increased expression of aquaporin-4 channels in glioma cells, and reduced expression of hypoxia-inducible factor 1α (HIF-1α) and signal transducer and activator of transcription 5B (STAT5B) in glioma cells [2, 8, 9, 10•, 11].

## Treatment options

As noted above, the overall frequency of seizure activity in brain tumor patients ranges from 35 to 70%, with seizures at presentation in 20–40% [1–5, 6•]. In another 10% of patients, seizures will be experienced at some point during the course of their illness. Before we review the use of antiepileptic drugs (AEDs) and pharmacological approaches in BTRE patients, it will be useful to briefly discuss the efficacy of non-AED—antitumor—approaches to seizure control.

### *Surgical resection*

The data seems clear that a gross total resection (GTR) versus a non-GTR in patients with gliomas, brain metastases, and meningiomas results in improved overall seizure control [2–5, 6•]. In addition, using a supra-total resection beyond the contrast-enhancing border of the tumor (i.e., into the surrounding FLAIR region) in GBM patients can result in improved seizure control and overall survival in comparison to routine GTR [17].

### *Radiotherapy*

The use of RT has been documented to improve seizure control in low-grade and high-grade glioma patients [2–5, 6•]. In a study of low-grade glioma patients, RT resulted in a 50% reduction of seizure activity in 56–77% of the cohort, with seizure freedom in 38–80%. In a series of high-grade and low-grade glioma patients, 77% of the cohort showed a 50% reduction in seizure frequency, with seizure freedom at 12 months in 38% of patients. A beneficial effect of RT on seizure control in patients with brain metastases has not yet been documented [18].

### *Chemotherapy*

It is well established that chemotherapy with alkylating agent-based regimens has the potential to improve seizure frequency, including temozolomide (TMZ), the combination regimen procarbazine, CCNU (lomustine), and vincristine (PCV), and CCNU monotherapy [2–5, 6•, 19]. In low-grade glioma patients, the use of TMZ has been associated with seizure freedom rates ranging from 13 to 50%, with similar rates for PCV in the 13–60% range. However, this effect is not as prominent in the setting of TMZ treatment of elderly GBM patients [20].

## Pharmacologic treatment

The general consensus among Neuro-Oncologists, Epileptologists, and Neurosurgeons is that after a single verified seizure—either witnessed or with an unequivocal history—a patient with a primary or metastatic brain tumor should be placed on an AED, and will be considered to have BTRE [2–5, 6•, 8, 21]. This is also the official position of the International League against Epilepsy (ILAE), as well as from an updated Society for Neuro-Oncology (SNO) and European Association of Neuro-Oncology (EANO) practice guideline [21, 22•]. Tumor histology, grade, location in the brain, and molecular markers do not play a role in determining if the patient should be placed on an AED, or which AED should be the first choice. Since most BTRE seizures are of the focal or partial type, the potential AEDs to consider should be approved for that indication. In addition, the efficacy of the available AEDs against BTRE events are fairly equivalent, so the final choice for the initial AED monotherapy will also be determined by patient-related factors, such as age, organ dysfunction, co-morbidities, concomitant drugs, and other therapies, as well as the side effect profile of the individual AEDs [2–5, 6•, 8, 21]. For patients with BTRE, it is best to avoid first-generation AEDs that are hepatic CYP3A4 enzyme inducers (EIAED), such as phenytoin, carbamazepine, and phenobarbital, due to the risk of compromising concurrent chemotherapy. Second-generation AEDs that are non-enzyme inducers (NEIAED) are usually the best choice for BTRE and include levetiracetam (LEV), lacosamide (LCM), lamotrigine, valproic acid (VPA), topiramate, and zonisamide (see Table 1). Of the available NEIAEDs to consider for initial monotherapy, the two with the most evidence for efficacy against focal epilepsy are LEV and VPA, which have Class 1A and 1B evidence, respectively.

**Table 1:** Overview of common antiepileptic drugs and select adverse effects

Drug	Mechanism of action	Usual starting dose	Usual maximum dose	Select adverse effects
Brivaracetam	Binds to synaptic vesicle 2a (SV2A) protein	50 mg twice daily	200 mg/day	Drowsiness, dizziness, sedation, psychiatric disturbance
Clobazam	γ-aminobutyric acid (GABA) potentiation	5–15 mg/day	80 mg/day	Drowsiness, lethargy, aggressive behavior, irritability
Lacosamide	Slow sodium channel inactivation	50 mg twice daily	600 mg/day	Drowsiness, nausea, headache, cardiac arrhythmias
Lamotrigine	Sodium channel blockade; some effect on γ-aminobutyric acid (GABA) potentiation	With enzyme inhibitor: 12.5 mg to 25 mg every other day With enzyme inducer: 50 mg/day Without interacting medications: 25 mg/day	With enzyme inhibitor: 200 mg/day With enzyme inducer: 500 mg/day Without interacting medications: 375 mg/day	Nausea, headache, insomnia, delayed hypersensitivity reactions, and rash including Stevens-Johnson syndrome (SJS)
Levetiracetam	Binds to synaptic vesicle 2a (SV2A) protein	500 mg twice daily	3000 mg/day	Drowsiness, dizziness, psychiatric symptoms (behavioral changes, depression, agitation)
Perampanel	Non-competitive antagonist of the inotropic α-amino-3-hydroxy-5-methyl-4-isoxazole propionic acid (AMPA) glutamate receptor	2 mg once daily	12 mg once daily	Neuropsychiatric effects (irritability, aggression, anxiety), dizziness, gait disturbance, weight gain
Valproic acid	Sodium channel blockade; γ-aminobutyric acid (GABA) potentiation	10–15 mg/kg/day	60 mg/kg/day	Somnolence, nausea/vomiting, thrombocytopenia, neutropenia, weight gain, osteopenia/osteoporosis, pancreatitis, hepatotoxicity
Zonisamide	Sodium and calcium channel blockade	100 mg/day	400 mg/day	Dizziness, drowsiness, psychiatric symptoms including depression, memory problems, and word-finding difficulties, blood dyscrasias, rash, nephrolithiasis

Adapted from the below references:

Briviact (brivaracetam) [prescribing information]. Smyrna, GA: UCB Inc; May 2023.

Onfi (clobazam) [prescribing information]. Deerfield, IL: Lundbeck; January 2023.

Lamictal (lamotrigine) [prescribing information]. Research Triangle Park, NC: GlaxoSmithKline; February 2023.

Keppra (levetiracetam) [prescribing information]. Smyrna, GA: UCB Inc; September 2022.

Fycompa (perampanel) [prescribing information]. Nutley, NJ: Eisai Inc; December 2022.

Depakote (divalproex sodium) [prescribing information]. North Chicago, IL: AbbVie Inc; February 2023.

Zonisamide [prescribing information]. Morgantown, WV: Mylan Pharmaceuticals Inc; July 2018.

Vimpat (Lacosamide) [prescribing information]. Smyrna, GA: UCB Inc; December 2023.

For grade 2–4 glioma patients with BTRE, there has been a recent meta-analysis by de Bruin and colleagues [23••] of the efficacy and tolerability of AEDs. They evaluated the outcomes data for AED monotherapy and polytherapy from 66 studies. In terms of the efficacy of monotherapy, the highest seizure freedom rate at 6 months was with phenytoin, while at 12 months LEV and pregabalin demonstrated the highest efficacy. For ≥ 50% seizure reduction rates, LEV was noted to have the highest efficacy at 6 and 12 months. In addition, LEV had the lowest treatment failure rate. When using polytherapy with follow-up ≥ 6 months, the most efficacious combinations were LEV with phenytoin or VPA. Lacosamide was also considered to be an excellent choice for add-on therapy in BTRE. This data is consistent with the results of a recent international survey of European Neuro-Oncology professionals, in terms of their AED preferences in brain tumor patients [24•, 25]. The vast majority of respondents prescribed an AED in the setting of BTRE for patients with gliomas (98%), meningiomas (85%), and brain metastases (90%). Levetiracetam was the first choice of AED in 90% of the respondents, due to the perception of it having the highest efficacy, a favorable adverse event profile, and lack of any significant interactions with antineoplastic treatments. Other choices for a trial of monotherapy or as an add-on AED were LCM, lamotrigine, and VPA. Surgical respondents were more likely to consider using an EIAED as initial therapy for BTRE in comparison to non-surgical respondents. In addition, surgical respondents were also more likely to consider a prophylactic AED in a brain tumor patient without a verified seizure.

### *AED prophylaxis in newly diagnosed brain tumor patients*

Several updated guidelines have been recently published addressing the issue of whether or not to consider the use of prophylactic AEDs in newly diagnosed brain tumor patients, including gliomas, meningiomas, and brain metastases [22•, 26, 27]. The recent SNO and EANO practice guideline update is consistent with older reports (e.g., American Academy of Neurology), that do not recommend starting an AED in a newly diagnosed brain tumor patient who has not had a verified seizure event [22•]. There is no evidence that AED prophylaxis will increase seizure-free survival or reduce the frequency of first seizures at 6 months from diagnosis. These conclusions are based on 3 randomized trials that provide class I evidence, as well as 8 class II studies. Similar conclusions have been reached for patients with meningiomas and brain metastases, although there is still controversy regarding prophylaxis in patients with metastases from melanoma [26, 27]. In addition, there is insufficient evidence for tumor location, extent of surgical resection, tumor histology or grade, imaging features, or molecular pathology to influence the decision regarding AED prophylaxis in patients with BTRE.

As noted above, in a brain tumor patient after a first verified seizure event, there is consensus to initiate an AED for an attempt at monotherapy—LEV is the first option in the majority of cases [1–5, 6•, 8, 21, 22•, 24•]. If the patient continues to have seizure episodes with initial monotherapy at maximally tolerated doses (which occurs in roughly 50–60% of glioma patients), and there has been some reduction in seizure frequency, then the recommendation would be to add on a second AED; the best initial options for an add-on AED are LCM, VPA, or lamotrigine. If the initial monotherapy did not result in any reduction in seizure frequency at maximum dosing, then one of the other AED options noted above should be started as monotherapy. Refractory BTRE is noted in 15% of GBM patients and 40% of grade 2 gliomas and is present when patients have not achieved seizure freedom after using two AEDs at maximally tolerated doses [1–5, 6•]. In this setting, it is common to add on a third AED; however, the additional benefit to seizure control has to be weighed against the likely increase in toxicity and side effects. In general, add-on AEDs should have a different mechanism of action to the primary anticonvulsant, in order to reduce the potential for intolerable side effects.

## Class of drugs

### Levetiracetam

As noted above, over the past 20 years LEV has been demonstrated to be a very effective AED for focal and generalized seizures in patients with primary epilepsy, as well as in patients with BTRE [1–5, 6•, 8, 23••, 24•, 28]. The anticonvulsant effect of LEV is mediated through binding to the synaptic vesicle protein SV2A, with subsequent modulation of neurotransmitter release. LEV demonstrates a bioavailability of 100% and has minimal protein binding of less than 10%. Metabolism of LEV occurs by enzymatic hydrolysis of the acetamide group, independent of liver function—therefore, no adjustments are necessary for hepatic diseases. Dose adjustments for renal disease are correlated with creatinine clearance and should be decreased for moderate to severe disease. The most common adverse reactions when using LEV are psychiatric—occurring in roughly 7–13% of patients, and include irritability, aggression, anxiety, depression, emotional lability, and psychosis [28, 29]. LEV is considered very “clean,” and has minimal interactions with other drugs, including chemotherapy and related antineoplastic therapies.

Several recent papers have further increased the perception that LEV should be the initial AED consideration in the majority of patients with BTRE; as long as there are no significant psychiatric pre-morbidities [1–5, 6•, 8, 23••, 24•, 30, 31]. In the first report from van der Meer and colleagues, a matched comparison was made between LEV (*N* = 429) and VPA (*N* = 429) for first-line treatment of BTRE in glioma patients [30]. The cumulative incidence of treatment failure rate for any reason at 12 months was significantly different between the groups: LEV = 33% versus VPA = 50%; *P* < 0.001. Similarly, when looking at reasons for treatment failure due to uncontrolled seizures, the rates at 12 months were significantly different: LEV = 16% versus VPA = 28%; *P* < 0.001. There were no differences noted for treatment failure due to adverse effects (14% vs 15%). The other report from the same group reviewed first-line AED treatment with LEV versus EIAEDs for BTRE in glioma patients [31]. The EIAED cohort had a significantly higher risk of treatment failure for any reason compared to the LEV group (aHR = 1.82; *p* = 0.005). The treatment failure rate due to uncontrolled seizures was similar between the EIAED and LEV groups (aHR = 1.32; *p* = .300). However, the rate of treatment failure due to adverse side effects was significantly higher in the EIAED group compared to the LEV cohort (aHR = 4.87; *p* = 0.001).

### Valproic acid

Valproic acid has a very broad spectrum of antiepileptic activity and is used worldwide [1–5, 6•, 8]. The mechanism of action is mainly thought to be related to increasing GABAergic activity in the brain, with a decrease in degradation and an increase in synthesis of GABA, resulting in a potentiation of postsynaptic GABAergic inhibition. There is also evidence for the activation of calcium-dependent potassium conduction. In adults, VPA demonstrates near full bioavailability at 90%, with a small volume of distribution. It is highly protein-bound at 90% and is eliminated mainly by hepatic biotransformation. Valproate has more potential for drug-drug interactions and adverse side effects than LEV, including idiosyncratic toxicities such as hepatic injury, pancreatitis, hyperammonemia, and thrombocytopenia.

As noted above, the most recent recommendation for the use of VPA in brain tumor patients with BTRE is in the setting of an “add-on” AED to LEV or lacosamide [2–5, 6•, 8, 23••, 24•, 30]. VPA is not recommended to be used as the primary AED for BTRE instead of LEV or lacosamide in the majority of patients. It does have excellent broad-spectrum AED activity, but has more drug-drug interactions and other potential toxicities that could limit its safety and tolerability.

### Lacosamide

Lacosamide was FDA-approved as an AED in 2008 and remains a Schedule Class V controlled drug in the USA [1–5, 6•, 8]. The main mechanism of action of lacosamide is via enhancement of slow inactivation of voltage-gated sodium channels. In addition, lacosamide interacts with the collapsing response mediator protein 2 (CRMP2), possibly inhibiting axonal sprouting that may underlie the progression reported in chronic epilepsy. Lacosamide has an excellent bioavailability of 100%, with low protein binding of less than 15%. It is metabolized mainly in the liver by isoenzymes CYP3A4, CYP2CP, and CYP2C19. Lacosamide and its metabolites are 95% excreted in the urine, with 40% unchanged drug. Dosing should be decreased in patients with hepatic disease and not used if there is severe liver dysfunction. No dosing adjustments are necessary in patients with mild or moderate renal disease. Similar to LEV, lacosamide has very minimal drug-drug interactions and does not induce the hepatic CYP3A4 enzyme system. However, it has a lower rate of neuro-psychiatric side effects than LEV.

As noted above, in recent years, lacosamide has been demonstrated to be an effective AED in brain tumor patients with BTRE—mainly as an “add-on” drug [1–5, 6•, 8, 23••, 24•, 30]. Several earlier papers evaluated the efficacy of lacosamide in BTRE, with positive results [32, 33]. Villanueva and co-workers [32] reported the results of an open-label observational study of adding lacosamide to 105 patients with BTRE who had a lack of efficacy or adverse events with prior AEDs. The addition of lacosamide resulted in a seizure-free rate at 6 months of 30.8%, with 66.3% of the patients having a ≥ 50% reduction in overall seizure events. The report from Ruda et al. [33] was an observational study of 71 patients with gliomas and BTRE with uncontrolled seizures, who had lacosamide added to their current AED regimen. They observed a seizure reduction of ≥ 50% at 3, 6, and 9 months of 74.6%, 76%, and 86.2%, respectively. In addition, at 3, 6, and 9 months, they noted seizure freedom rates of 42.2%, 43%, and 50%, respectively. The seizure reduction ≥ 50% rates and seizure freedom rates were higher in patients who received lacosamide as the first add-on AED.

More recent reports are also consistent in demonstrating the efficacy of lacosamide in patients with BTRE [34–36]. The report from Ruda and colleagues [34] describes the results from a European multicenter study of add-on lacosamide to patients with low-grade gliomas and resistant BTRE. They evaluated 79 patients who completed the study, who at 6 months had a ≥ 50% responder rate of 76.7%. At 6 months, 34.9% of the cohort was seizure-free. Improvements in the Patient’s Global Impression of Change scale were noted in 64.5% of patients. In addition, the Kaplan-Meier estimated 6-month retention rate while on lacosamide was 86%. In another European multicenter study, van Opijnen and co-workers [35] reported the results of a trial comparing the efficacy of lacosamide and lamotrigine as add-on therapy in 139 glioma patients with BTRE who had failed first-line treatment with LEV or VPA. At 12 months, the cumulative incidence of treatment failure for any reason was not significantly different between the lacosamide and lamotrigine cohorts—30% versus 38%, respectively. The cumulative incidence of treatment failure due to uncontrolled seizures was similar for lacosamide and lamotrigine—11% versus 18%, respectively. Adverse events for both drugs were also noted to be equivalent. In a multicenter study by the Italian group, lacosamide was used as monotherapy in a group of patients with primary brain tumors and BTRE [36]. The use of lacosamide resulted in seizure freedom in 64.4% of the cohort at 3 months and in 55% of patients at 6 months. Seizure control was better in patients who initiated AED therapy with lacosamide, in comparison to those who had one or two other AEDs prior to starting lacosamide monotherapy. The discontinuation rate for lacosamide was very low at 1.5%. Overall, lacosamide is an excellent choice for add-on AED therapy in patients who are not seizure-free while on initial LEV or VPA treatment. In select patients, it can also be considered for AED monotherapy.

### Brivaracetam

Brivaracetam (BRV) is recently licensed in Europe and the USA for the treatment of partial-onset seizures in patients ≥ 1 month of age with epilepsy as monotherapy or adjunctive therapy [6•, 8, 37, 38]. It is the n-propyl analogue of LEV and also acts as a high-affinity ligand for the synaptic vesicle protein SV2A. SV2A is an integral transmembrane glycoprotein expressed in neurons, which is involved in the modulation of synaptic vesicle exocytosis and neurotransmitter release. BRV binds to SV2A with a 15–30-fold higher binding affinity than LEV, and may act at a different binding site or during different conformational states of the protein. BRV crosses the BBB more rapidly than LEV and reaches maximal brain concentration within minutes of an intravenous bolus. After oral administration, BRV is absorbed rapidly and is unaffected by the presence of food, except for high-fat meals, which can delay absorption. The drug undergoes first-order pharmacokinetics and has a low plasma protein binding of 17.5%. The major route of metabolism is via hydrolysis of the acetamide group, with the production of a carboxylic acid metabolite. BRV has been shown to have low potential for drug-drug interactions. The efficacy and tolerability of adjunctive BRV have been demonstrated in six randomized, placebo-controlled clinical trials of patients with refractory epilepsy [37, 38]. These trials and subsequent meta-analyses have been consistent in showing that BRV is significantly more effective than placebo at reducing seizure frequency by ≥ 50%, as well as for being able to induce seizure freedom. BRV is generally well tolerated and has a lower incidence of psychiatric disorders than LEV [29].

BRV has only recently been applied to brain tumor patients with refractory BTRE [6•, 8, 39]. Maschio and co-workers [39] evaluated 33 patients with refractory BTRE in an Italian multicenter study, including low-grade gliomas (*N* = 11), high-grade gliomas (*N* = 5), glioblastomas (*N* = 10), meningiomas (*N* = 6), and one primary brain lymphoma. They were all treated with add-on or replacement BRV at a starting dose of 25 mg/day and then titrated upwards to a mean dose of 175 mg/day. The mean seizure frequency in the month preceding the onset of BRV was 7.0, while after treatment with BRV, the mean seizure frequency was reduced to 2.0 (*p* = 0.001). Seizure freedom was noted in 20 patients (60%), while another six had a reduction of ≥ 50% (18.1%). The responses were similar between patients in whom BRV replaced LEV, versus being added to other non-LEV AEDs. This preliminary data suggests that BRV has efficacy as an add-on AED in patients with BTRE, and may also be a good substitute for LEV in the setting of psychiatric side effects or lack of efficacy.

### Lamotrigine

Lamotrigine was FDA-approved in 1994 as an AED for the treatment of partial-onset seizures and primary generalized epilepsy as monotherapy or as an add-on anticonvulsant in adults and children [1–5, 6•, 8, 40]. The main mechanism of action of lamotrigine is thought to be the inhibition of voltage-sensitive sodium channels, although calcium channels may also be a target. Lamotrigine has excellent bioavailability of 98% and protein binding of 55%. The metabolism occurs by glucuronic acid conjugation. Dosing usually starts at a reduced amount (50–100 mg bid) and is slowly titrated upwards to reduce the risk of severe cutaneous and other hypersensitivity reactions, with a maximum of 400 mg/day. Lamotrigine is not a hepatic enzyme-inducing drug. However, its metabolism can be influenced by other medications that induce the cytochrome P450-3A4 system.

The application of lamotrigine to brain tumor patients with refractory BTRE has been limited [6•, 8, 23••, 24•, 35]. As noted above, the European multicenter study by van Opijnen et al. [35] involved comparing the efficacy of lacosamide and lamotrigine as add-on therapy in glioma patients with BTRE who had failed first-line treatment with LEV or VPA. At 12 months, the cumulative incidence of treatment failure for any reason was similar between the two cohorts. In addition, the cumulative incidence of treatment failure due to uncontrolled seizures was equivalent for lacosamide and lamotrigine. Therefore, limited preliminary evidence would suggest that lamotrigine should be considered a reasonable option for the add-on treatment of refractory BTRE.

### Perampanel

Perampanel was approved by the FDA in 2012 as a non-competitive AMPA receptor antagonist that can be used for the treatment of focal-onset seizures with or without secondary generalized seizures, as well as primary generalized tonic-clonic seizures, and is a Schedule Class III controlled substance due to its abuse potential [41, 42]. The main mechanism of action of perampanel is via non-competitive antagonism and inhibition of AMPA-glutamate receptors, which have been shown to be a mechanism of epileptogenesis and overexcitability in peritumoral neurons [9, 10•]. Perampanel has excellent bioavailability of 100% and extensive protein binding of 95%. It is metabolized by the CYP3A4 and CYP3A5 hepatic enzymes. Dosing should be modified for mild and moderate hepatic disease; it should not be used in severe hepatic impairment. It is also not recommended for use in patients with severe renal disease.

There have been several studies reported using perampanel as an add-on AED in patients with refractory BTRE; no studies have attempted monotherapy [6•, 8, 23••, 43–47]. The 6-month seizure freedom rate of polytherapy ranged from 31 to 60%, with a weighted average of 41%. The 12-month seizure freedom rate was roughly 45%. Seizure reduction rates ≥ 50% at 6 and 12 months were 92% and 82%, respectively. Overall treatment failure rates with polytherapy that included perampanel ranged between 0 and 17%.

### Clobazam

Clobazam is a 1,5-benzodiazepine agonist that was synthesized with the goal of having increased efficacy and less sedation, and received FDA approval in 2011; it remains a Schedule Class IV controlled substance in the USA [6•, 8, 48]. The main mechanism of action of clobazam is to modulate GABA-induced chloride influx via binding to the benzodiazepine receptor on GABA_A_ channels. It has a greater selectivity for anxiolytic and antiepileptic subunits, versus those involved in mediating sedation. Clobazam has a bioavailability of 90–100% and significant protein binding of 80–90%. The metabolism is mainly in the liver by the CYP3A4 isoenzyme. There has been limited application of clobazam to brain tumor patients with refractory BTRE [6•, 8, 23••, 48]. Brahmbhatt and colleagues [48] used clobazam as an add-on therapy in a series of 35 patients with primary brain tumors and persistent seizure activity. A positive response to clobazam was defined as a ≥ 50% reduction in seizure frequency. In 33 evaluable patients, they noted positive responses in 31 patients (93.9%). In 10 patients (30.3%), seizure freedom was achieved within 6 months of initiating add-on clobazam. The authors concluded that clobazam had efficacy as an add-on AED in patients with refractory BTRE.

### Zonisamide

Zonisamide has been FDA-approved as adjunctive therapy for partial-onset seizures in the USA since 2000 [2, 8]. The main mechanisms of action are a blockade of sodium channels and a reduction of voltage-dependent transient induced currents (i.e., T-type calcium channels). Zonisamide has a bioavailability of 90% and protein binding of 40%. Zonisamide undergoes metabolism by CYP3A4 and is then excreted in the urine. Zonisamide has had minimal application to patients with refractory BTRE thus far. In an updated study, Maschio and co-workers [49] treated 9 evaluable patients with zonisamide add-on therapy. The pre-treatment mean weekly seizure frequency was 3.2, while at the final follow-up, the mean weekly seizure frequency had been reduced to 0.18 (*P* = 0.05).

## Data Availability

No datasets were generated or analysed during the current study.
